# Advanced strategies for nucleic acids and small-molecular drugs in combined anticancer therapy

**DOI:** 10.7150/ijbs.79328

**Published:** 2023-01-01

**Authors:** Chong Qiu, Yanyan Wu, Qiaoli Shi, Qiuyan Guo, Junzhe Zhang, Yuqing Meng, Chen Wang, Fei Xia, Jigang Wang, Chengchao Xu

**Affiliations:** 1 Artemisinin Research Center, and Institute of Chinese Materia Medica, China Academy of Chinese Medical Sciences, Beijing 100700, China.; 2 Chinese Academy of Medical Sciences & Peking Union Medical College, Beijing 100730, China.; 3 School of Traditional Chinese Medicine, Southern Medical University, Guangzhou 510515, China.; 4 College of Integrative Medicine, Laboratory of Pathophysiology, Key Laboratory of Integrative Medicine on Chronic Diseases, Fujian University of Traditional Chinese Medicine, Fuzhou 350122, China.; 5 Department of Nephrology, Shenzhen key Laboratory of Kidney Diseases, and Shenzhen Clinical Research Centre for Geriatrics, Shenzhen People's Hospital, The First Affiliated Hospital, Southern University of Science and Technology, Shenzhen 518020, China.

**Keywords:** anticancer, nucleic acids, small-molecular drugs, combination, synergistic therapy

## Abstract

Cancer has been considered as complex malignant consequence of genetic mutations that control the cellular proliferation, differentiation and homeostasis, thus making tumor treatment extremely challenging. To date, a variety of cargo molecules, including nucleic acids drugs (pDNA, miRNA and siRNA), therapeutic drugs (doxorubicin, paclitaxel, daunomycin and gefitinib) and imaging agents (radioisotopes, fluorescence dyes, and MRI contrast agents) have been regarded as the potential medicines in clinical application. However, non-single therapeutic drug could induce the satisfied clinical results because of tumor heterogeneity and multiple drug resistance and the nanotechnology-based combined therapy is becoming an advanced important mode for enhanced anticancer effects. The review gathers the current advanced development to co-deliver small-molecular drugs and nucleic acids for the anticancer therapy with nanomedicine-based combination. Furthermore, the superiority is definitely presented and the barriers are detail discussed to surmount the clinical challenges. In final, future perspectives in rational direction for combined tumor therapy of drugs and nucleic acids are exhibited.

## Introduction

Cancer is a malignant type of genetic disease in which not one, but several, mutations are required. It continues to be main factors of death with increasing incidence throughout the world, accounting for over eight million people worldwide annually according to the worldwide scientific report published in 2022 1, which equated to the entire population of New York. Therefore, it has been regarded as the extremely urgent that exploring the reasonable treatments to ameliorate or even surmount the current situation.

Significant advances in anticancer therapy have been made in recent decades. Of the various therapeutic agents, chemotherapy has become a preferred choice for most managements of cancers due to its high cytotoxicity of chemo agents against cancer cells 2. However, these conventional small-molecular anticancer drugs usually show inherent defects (such as the poor bioavailability, non-specific distribution, rapid blood clearance and multiple drug resistance in physiological conditions), often resulting in severe side effects in patients (sudden death, gastric bleeding, alopecia and heart failure). To improve these limitations, several nano-delivery approaches have been introduced, such as liposomes, nanoparticles and so on 3. Some of these studies indeed showed an increase in drug accumulation and a decrease in cytotoxic side effects in tumor tissues, some of which have been already in clinical trials 4. Nevertheless, the major of additional materials may inevitably bring out adverse impacts, such as, the drug loading capacities were relatively low, most delivery materials which were just as excipients may cause added toxicities or undesirable immune response 5, 6.

As genetic aberrations involved in tumor cell signaling are further explored, nucleic acids have been probed as functional bio-macromolecules applied to the treatment of cancer 7. Gene drugs (such as siRNA, miRNA etc.), compared to the small-molecular drugs, could effectively augment down-regulated the target mRNA or knockdown overexpressed proteins, which have been regarded as the attractive approach for suppressing tumor cell growth and invasion 8, 9. However, these bio-macromolecules still bear several non-negligible shortcomings to hinder the utilization in clinic. For example, nucleic acids were opted to be degraded by RNase families; have rapid clearance and poor cellular uptake etc. Appropriate nano-delivery materials have been emerged to accelerate their clinic development, which displayed that the loaded nucleic acids could be effectively protected and released at the desired intracellular site 10.

**Figure 1:**
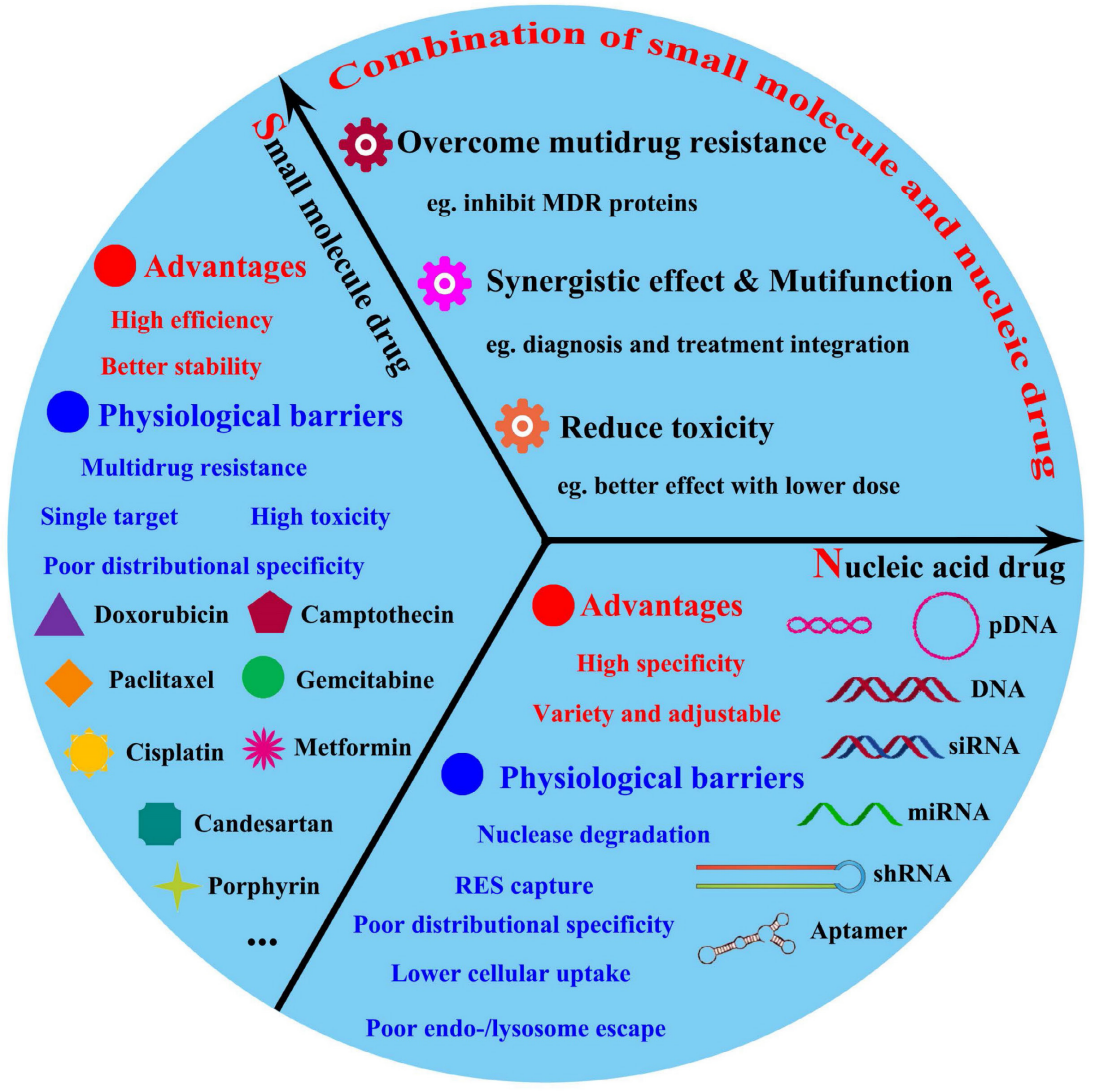
The major advantages and physiological barriers of small molecule drugs/nucleic acid drugs therapy and the advantages offered by combined them.

Up to present, it has been indeed achieved in substantive progress for cancer treatment by either chemotherapy small-molecular drugs or nucleic drugs; nevertheless, this malignant disease was still obstinate to be eliminated. This predicament may be attributed from the molecular and structural complexity of tumor, which cancers were regarded as various genetic disorders involved in multiple cells signaling 11-13. So, it is possible that a single therapeutic strategy is insufficient to halt the progression of most cancers. Therefore, nanomedicine-based combination anticancer therapy between nucleic acids drugs and small-molecular drugs, which targeted at different molecular levels of dysregulated tumor cellular signaling pathways, could be expected to break through the current dilemma in tumor treatment field. Actually, the co-therapy of small-molecular drugs and nucleic acids has displayed the promoted anticancer effects and become the promising potential for tumor treatment 14-16. As shown in **Figure 1**, this review firstly will cover an overview of advanced developments of combined-therapy that simultaneously deliver both chemotherapy drugs and nucleic acids to tumor targets; subsequently, elaborate these significant design parameters that were essential for the co-administration of small-molecular drugs and nucleic acids drugs (pDNA, siRNA and miRNA); besides, the potential merits and shortcomings will be respectively discussed; lastly, future perspectives in rational direction for combined tumor therapy of drugs and nucleic acids will be exhibited.

## Different modes of drug combination

**Figure 2:**
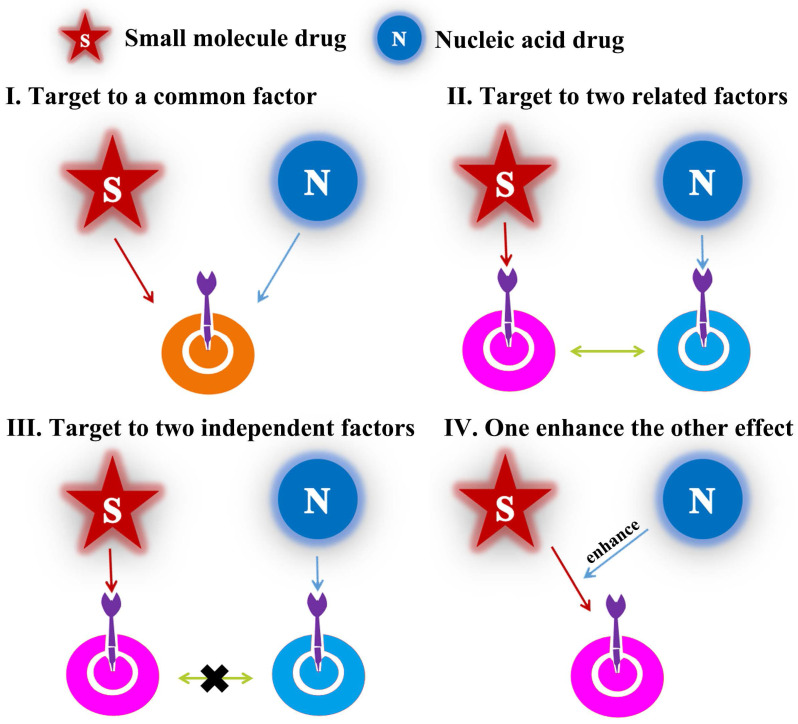
Structural illustration of four synergetic mechanisms of combined-therapy.

As mentioned above, the emergence of cancer was generally accompanied of multiple mutations of numerous key proteins which regulated the growth and metastasis of cancer cells. Therefore, in combination with small-molecular drugs, nucleic acids could act another prominent role by down-regulation/knockdown of targeted RNA/proteins in cancer cells. Over the last two decades, combined-anticancer therapy between chemo-and gene drugs have become an increasingly strategy either in preclinic or clinic, which about 1340 articles have been reported according to incomplete statistics. In this review, we covered almost researches and generalized the different synergistic mechanisms. There were divided into four categories (**Figure 2**): (1) the chemotherapy drugs and nucleic acids shared the same target point, such as** Figure 2I**, which may importantly enhance the therapeutic effect, for example, anti-neovascularization, apoptosis induction or anti-angiogenesis; (2) the two drugs not acted a target but cooperatively associated in one signaling pathway, like as** Figure 2II**, which may obtain the unexpected results by similar to “Butterfly Effect”; (3) the two ones coped with different targets, for example,** Figure 2III**; (4) one drug could have an auxiliary reinforcement for the other, for instance, **Figure 2IV**. Among the four classifications, the second one, which attempted to actualize multiple-targeted anticancer therapy, occupied most of the proportion (~60%) in recent studies; besides, the third class mainly involved to the integration of diagnosis and treatment for cancer. Actually, all these synergistic mechanisms embodied the unique advantages of combined-therapy, which gave the credit to the assembly structures/modes of nano-medicines encapsulated by nucleic acids drugs and small-molecular drugs.

## Different types for drug encapsulation

In the past two decades, on account of the emergence of systemic toxicity and multiple drug resistance (MDR) for small-molecular drugs 17, the development of nanomedicine-based combined anticancer therapy between nucleic acids and chemotherapeutic drugs has paralleled the growth of gene nanotechnology. The combined treatment could importantly increase its therapeutic efficacy in many kinds of cancer. The disadvantages associated with chemotherapy could be mitigated by the addition of gene drive drugs; furthermore, the efficacy of gene therapy could also be strengthened by small molecule drugs with more potent effects. All of which led to the significant increase in treatment for aggressive cancers, where the progression and invasion involved various physiologic or pathologic factors 18. Therefore, construction for a nano-medicine should be prerequisite for perfectly exerting the synergistic effects, which could not only effectively load different drugs but also safely delivered these cargoes into the targeted sites. There were multiple nano-devices applied in the combined anticancer therapy, which could be divided into two categories according to different administration modes (**Graphical abstract**): (1) **“1+1” type**: separated encapsulation for combination therapy that free chemotherapeutics drug + gene loaded by nanocarrier or loading two agents into two individual nanocarrier; (2) **“2 in 1” type**: co-encapsulation for combination therapy including the corporate drug pairs (CDP), “co-assembling drug” with independent agents and drug-carrier conjugates delivering drug (DCDD).

### “1+1” type: separated encapsulation for combination therapy

One of the greatest challenges in the process of co-delivery of chemotherapeutics and gene drive agents is optimizing the timing/location of delivery of two drugs into tumor cells. In some cases, the addition of drugs will affect the pharmacokinetics of nano carriers and require major modifications to the design of nanocarriers, which will lead to the difficulty of delivering nucleic acids and small molecule drugs together in a single nanocarrier. Therefore, differences in toxicity profiles or optimal routes of administration often require the two agents to be formulated individually for separate administration so as to achieve the therapeutic better effects for combination anticancer therapy.

**Figure 3:**
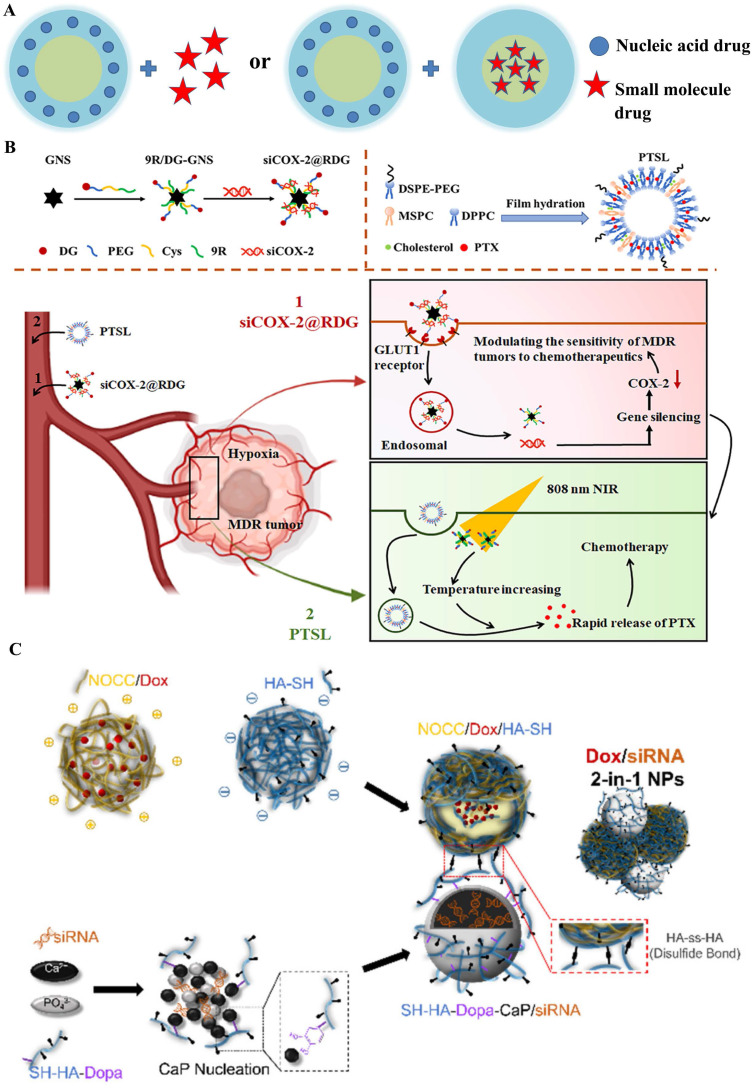
A) Structural illustration of co-delivery systems of “1+1” type. B) The sequential dual delivery systems based on siCOX-2@RDG and PTSL to overcome the hypoxia-induced MDR 22; C) The “2-in-1” integrated nanocarrier constructed from Dox-loaded NOCC/HA-SH NPs and SH-HA-Dopa-coated CaP/siRNA NPs linked through disulfide bond (HA-ss-HA) 23.

Based on this concern or principle, the sequential delivery of therapeutic drugs and nucleic acids to cancers has been increasingly adopted by researchers with great success in recent years, as shown in **Figure 3A**. One main manner of sequential delivery is combination of free chemotherapeutics drug and gene loaded by nanocarrier and it has the advantage of easily optimizing doses of both therapeutic agents. In the earliest works, Zhang et al. 19 performed a study in which rats were given a single injection of chloroquine two to three hours prior to delivery of polylysine-molossin/DNA via the portal vein and bile duct pathways to increase transfection efficiency. On the basis of that the hTNF-α could sensitize the cancer cells to chemotherapeutic drugs, SR Murugesan and co-workers 20 used gemcitabine to deal with Human pancreatic cancer cells after three hours indications of AdEgr.TNF.11D. The combination treatment was well tolerated, highly active, and resulted in marked delays in the growth of human pancreatic xenograft tumors compared to either agent alone in the in vitro experiments. However, the combination of free chemotherapeutic drug and gene loaded nanocarrier has some weaknesses: 1) it is difficult to deliver controllable ratios of drug to nucleic acid in the same target cells; 2) difficulty in synchronizing pharmacokinetics and biodistribution; and 3) unwanted toxicity in normal tissues caused by free drugs, leading almost to its conclusion.

Compared to the first manner, the other manner for sequential delivery that loading two agents into two individual nanocarrier (same or different) obtains more advantages: 1) independent controllable releasing time and rate of drug action; 2) achieve multiple therapeutic targets; and 3) avoid the toxicity of free agents, and it has been widely used to achieve the combination of two drugs, particularly for overcoming the tumor MDR under the fourth collaboration mechanism (**Figure 2IV**). Hence, Su et al. sequential inject G3-HD-OEI/TNFα gene vector and liposomal doxorubicin (DOX) into the tumor-bearing mice in order to delay the tumor growth in subcutaneous murine neuroblastoma Neuro2A 21. Huang et al. 22 fabricated a dual sequential delivery system composed of two independent nanosystems: cyclooxygenase-2 siRNA in poly-d-arginine (9R)/2-deoxyglucose (DG)-loaded gold nanostar (GNS) and paclitaxel-loaded thermosensitive liposome (PTSL), which has been proposed to overcome MDR mediated by hypoxia in tumors (**Figure 3B**).

Similarly, Wu et al. 23 designed a redox/pH-sensitive chimeric nanoparticles for co-delivering doxorubicin (DOX) and siBcl-2 in the HeLa cells. The integrated nanosystem was formed with two individual components through a redox-responsive thiol-disulfide bond: DOX loaded N, O-carboxymethyl chitosan (NOCC) complex with a thiolated hyaluronic acid (HA-SH) nanocarrier and dopamine (Dopa)-conjugated thiolated hyaluronic acid (SH-HA-Dopa)-coated calcium phosphate (CaP)-siRNA nanocarrier (**Figure 3C**). A key to the clinical realization of these treatments would be the development of a nanocarrier system allowing the simultaneous delivery of chemotherapeutic agents and nucleic acids to cancer, in which "co-delivery" would take place of combinatorial delivery.

**Figure 4:**
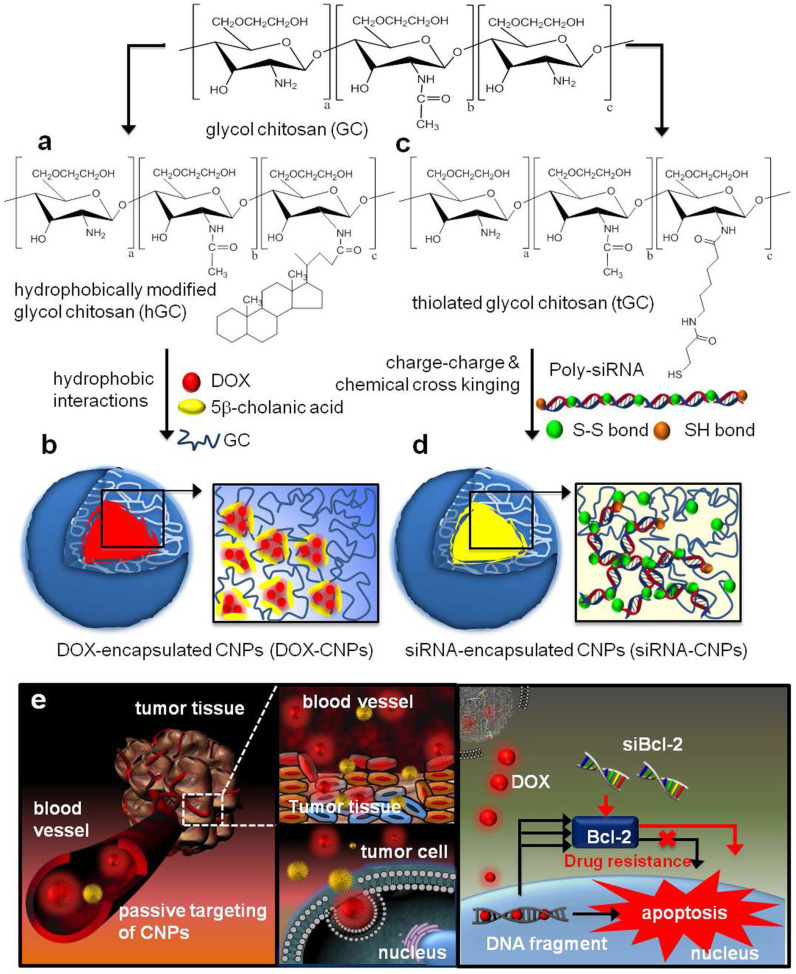
Structural illustration of co-deliver DOX and Bcl-2 siRNA by tumor-homing and biocompatible glycol chitosan (GC)-based nanocarriers (CNPs) to achieve optimal efficacy 26.

Moreover, encapsulation of different kinds of drugs into the same nanocarriers often possess more advantages: 1) could overcome different instability and in vivo behaviors (pharmacokinetics, tumor accumulation tendency); 2) lower the influence of the intrinsic physicochemical properties from different agents. Wang and co-workers developed a sequential treatment strategy with RGD-modified liposomes containing P-gp siRNA or DOX in order to improve MDR cancer therapy 24. In vivo studies of drug resistant MCF7/A tumor demonstrated significantly greater tumor growth inhibition followed by sequential treatment of RGD modified liposomes containing siRNA or DOX when compared to liposomal DOX treatment alone. Similarly, Shanthi Ganesh et al. 25 adopted the combination treatment of cisplatin and siRNAs encapsulated in CD44-targeting hyaluronic acid (HA)-based self-assembling nanocarriers. After the last 3 days of intra-articular HA-PEI/PEG/survivin siRNA injection, the HA-ODA/PEG/cisplatin was injected, which reversed cisplatin resistance and significantly retarded tumor growth (growth inhibition increased from 30% to 60%) in cisplatin resistant tumors. As shown in **Figure 4**, Hong et al. designed the DOX loaded CNPs (DOX-CNPs) by hydrophobic interactions and Bcl-2 siRNA loaded CNPs (siRNA-CNPs) by charge-charge interactions 26. Significantly, sequential treatment of DOX-CNPs and siRNA-CNPs resulted in significantly longer-term inhibition of tumor growth than each treatment alone. In addition, various nanocarriers also developed to overcome MDR, such as siRNA and paclitaxel co-delivered by triblock copolymer 27 or multifunctional nanocomplex 28, respectively, self-assembling nanoparticle system of siRNA and cisplatin 29, 30, and some other inorganic nanoparticles, such as graphene oxide for DOX and siRNA 31.

The basis of many drug-nucleic acid combinations is to first modify signaling pathways in cancer cells to render them sensitive to chemotherapeutic drugs, so the most important thing is the need of sequential release rather than the must of separate systems. Li et al. formed the sequential drug delivery system SiO_2_@AuNP, which featured step-by-step controlled and sequential delivery of siRNA, DOX, and HCPT in order to maximize its anticancer efficacy 32. This suggested that the sustained SiO_2_ release characteristics made the difference in T_max_ between HCPT and DOX about 8 to 12 h, and this increased the sensitizing efficacy of HCPT on DOX compared to co-administration (~10-fold). Similarly, the triblock copolymer (PCL-PEG-PHIS) was synthesized with folate-PEG-PHIS in order to construct a multifunctional targeted (PTX/siVEGF-CPPs/TMPM) polymer micelle for the sequential delivery of siVEGF-CPPs (disulfide bond-linked siVEGF and cell-penetrating peptides) and paclitaxel 33. Other sequential therapy, such as redox-sensitive glucolipid nanoparticle separately delivering siRNA and DOX to overcome MDR 34, PEI-grafted graphene oxide delivering siRNA and anticancer drugs sequentially 35 and so on. Above all, these sequential delivery nanocarriers show potential as a better effective co-delivery method for nucleic acids drugs and chemo drugs to enhance the efficacy of anticancer therapy.

However, under no circumstance can we ignore the fact that distinct assembly methods may produce the diverse bio-behaviors (like as drug release rate, onset time and biodistribution etc.) of small-molecular drugs and nucleic acids. Briefly, in order to maximize combined therapeutic efficacy, a proper co-delivery way and the proper combination of several drugs should be key factors for anticancer therapy. Meaningfully, co-load of therapeutic chemo drugs and nucleic acids drugs into one single nanocarrier (“2 in 1”) will offer various benefits based on synchronized pharmacokinetics, convenience and delivery of controllable relative amounts of both agents to target cells, which often is what the type “1+1” couldn't possess.

### “2 in 1” type: co-encapsulation for combination therapy

The concept of “co-delivery” implied that all different drugs were encapsulated in one nanocarrier. As we mentioned before, compared to the co-administrations by separate vectors, the “2 in 1” strategy could provide a guarantee that both the small-molecular drug and nucleic acids were synergistically introduced into the same tumor cells to promote the anticancer therapy; besides, it ensured that these drugs have a synchronous pharmacokinetics and tuned the reasonable proportions of administration dosage. Three subdivisions were presented and discussed their characteristics respectively in the next context.

#### Co-encapsulation following drug-drug interaction

**Figure 5:**
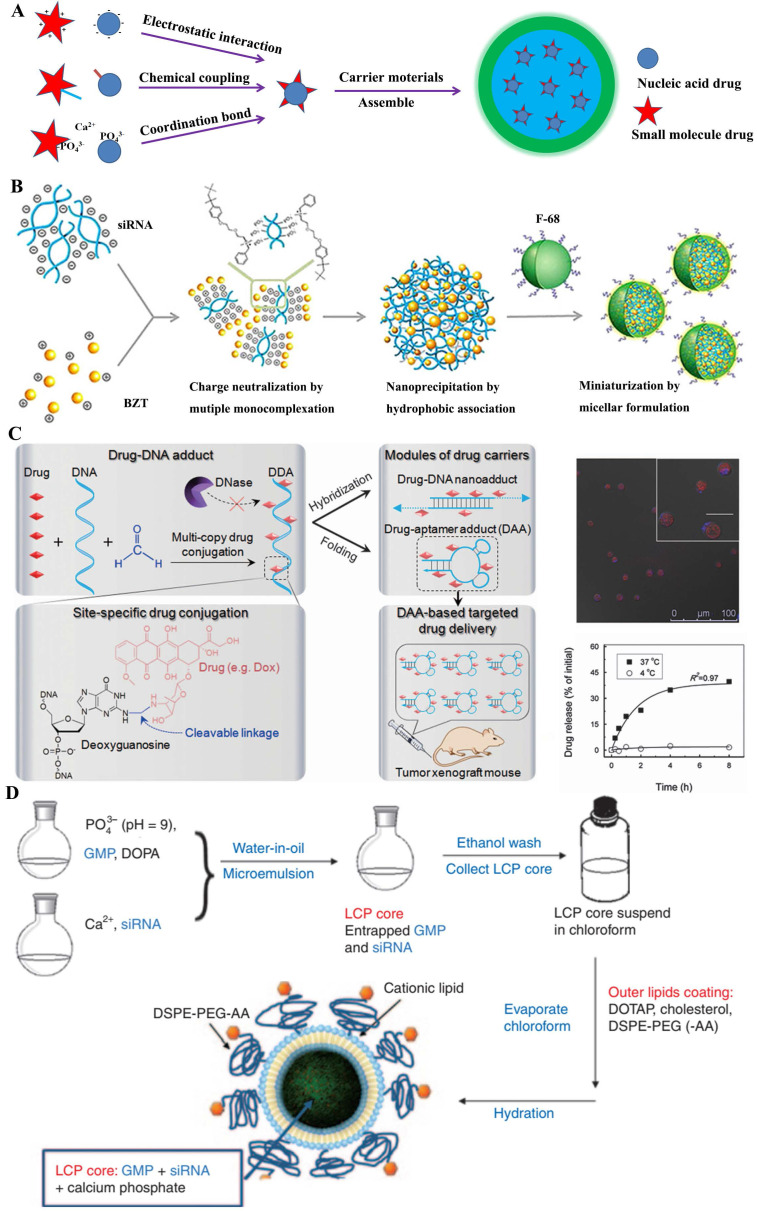
A) Structural illustration of co-encapsulation following drug-drug interaction; B) Ternary HMplexation containing benzethonium chloride (BZT) and siRNA 37; C) Nuclease-resistant synthetic drug-DNA adducts as a platform for anticancer therapy 38; D) The preparation method of GMP- and/or VEGF siRNA-loaded LCP nanoparticles 39.

Several researches displayed that both the small-molecular drug and nucleic acid were jointly encapsulated in the core of nano-delivery systems 36, which gave a more protection drugs in delivering progress, especially nucleic acids, and were synchronously released into cells. Generally, the formation of these nanomedicines was firstly constructed the corporate drug pairs (**CDP**) (**Figure 5A**), which required certain interactions between small-molecular drug and nucleic acid, like as electrostatic adsorption, coordination bond or covalent coupling; then a biocompatible skeleton molecule (e.g., polymers, lipids) were covered on the **CDP** surfaces to obtain the nano-system loading drugs.

Some traditional anticancer drugs comprised of the amino groups, like as doxorubicin hydrochloride (DOX), benzethonium chloride (BZT) and gemcitabine hydrochloride (GEM), electrostatically coupled with nucleic acids (siRNA or miRNA) by their negative phosphate backbone. Actually, employing the functional nucleic acids could boost the chemo-sensitization of cancer treatment with anticancer drugs. As was known, the overexpression of apoptotic genes NF-kB and Bcl-2 were observed in tumor invasion and metastasis, which has been regarded as the potential target for cancer treatment 40. M. Yousefie and colleague constructed a chitosan-based nanoparticle where co-delivery of doxorubicin and IL17RB-targeted siRNA for enhanced anticancer efficacy in breast cancer cells 41. Utilized the negatively charged carboxymethyl dextran (CMD), they designed chitosan-based nanoparticles containing CMD to effectively compress and encapsulate the DOX/oligonucleotide pairs, which significantly inhibited the growth and proliferation of cancer cells. Similarly, combining Bcl-2-targeted siRNA with anticancer drugs could promote the synergistic effect for tumor treatment. S. Kim and colleague 37 attempted to develop a more clinically relevant formation for small-molecular drugs and nucleic acids. Using a single complexation preparation, a colloidal system of hydrophobically associated multiple monocomplex (HMplex) was designed to co-delivery between Bcl-2 targeting siRNA and a monocationic anticancer drug (benzethonium chloride, BZT) (**Figure 5B**). Chosen the Bcl-2 protein overexpressed cells (MDA-MB-231) as the model, the assay data showed a dramatic reduction of Bcl-2 expression (down to 20%) with these co-delivery nanomedicines; remarkably, the level of early apoptosis was significantly increased from 6% to 18%, which implied that the siRNA could sensitized the resistant cancer cells and then enhanced the anticancer effect of BZT. In addition, R. Paulmurugan et. al. 42 selected the gemcitabine hydrochloride and antisense-miRNA-21 as the **CDP** encapsulated in a PLGA-PEG-based nano-device to treat hepatocellular carcinoma by the double emulsion method. In their research, the co-delivery system was shown to result in the 14% apoptotic cells in both Hep3B and HepG2 cells, and efficiently blocked endogenous miRNA-21 function and promoted the expression level of the PTEN target protein 39.

In fact, fluorescent tracers were customarily coupled with nucleic acids by covalent conjunction. These **CDPs** could be applied to both diagnosis and treatment for cancer, which was recently advocated by clinicians. As known, the magnetic resonance imaging (MRI), which was widely used to diagnosis in clinic, had the innate limitation of spatial resolution (ca. 100 mm) to be insatiable to monitor the intracellular transfection of nucleic acid drugs 43. J. Cheon and his colleague designed a multi-functional probe for simultaneous siRNA delivery and multimodal image in tumor treatment 44. In this nano-device structures, manganese-doped magnetism engineered iron oxide covered by bovine serum albumin (BSA) was selected to core section, where the **CDP** of fluorescent dyes (Cy5)/siRNA was conjugated by redox-responsive disulfide bonds; besides, the PEGylated RGD was also incorporated onto the surfaces to enhance special uptake by the ανβ3 integrin recognition. Several assay results showed that these nanomedicines could be clearly visualized in MDAMB-435 cells, which expressed ανβ3 integrin as breast-cancer cells; moreover, these loaded siRNAs displayed an important decline (~ 40%) of targeted GFP proteins at the low dose of 4 and 6 pmol. Similarly, Zhu et al. 38 reported the synthetic drug-DNA adducts (DDAs) for anticancer therapy (**Figure 5C**). Site-specific conjugation of multiple copies of anthracycline drugs was performed on each DNA, allowing programmable DNA and drug design for DDA preparation. DDAs were resistant to nucleases and stable for storage, but could release drugs gradually at physiological temperature. DOX-aptamer adducts significantly inhibited the xenograft tumor growth, which indicated the potential of DDAs for scale-up production and clinical application in anticancer therapy. Apart from this, aptamer-drug conjugates (ApDCs) also were potential application 45.

Except the methods mentioned above, ion coordination was also a driving force for **CDP** formation between small-molecular drug and nucleic acid. In general, the chelated phenomenon was based on the special recognition of non-toxic mrtal ions (e.g., Ca^2+^, Zn^2+^ ion) with phosphate bonds 46. Lipid/calcium/phosphate (LCP) nanoparticle could effectively deliver gene drugs into targeted cells 47. Based on this preparation mechanism, L. Huang and colleague 39 explored the co-delivery strategy of gemcitabine monophosphate (GMP) and VEGF-targeted siRNA for non-small-cell lung cancer (NSCLC) therapy (**Figure 5D**). They tried to utilize the co-delivery to act the different mechanisms for comprehensively proliferating tumor cells. As we know, GMP could induce apoptosis and the VEGF siRNA could block the tumor neovasculature by inhibition of the VEGF-VEGFR signaling. Their results showed that the combined-nanomedicine triggered a significant amount (~30%) of apoptotic cells, compared to the GMP nanoparticles (~12%). Zinc was another coordinate ion as key element for binding to nucleic acids 48, 49. For instance, Zn^2+^-dipicolylamine (Zn-DPA) derivatives, which bear the special selectivity for phosphate-containing molecules, were usually regarded as special probes for the membrane surfaces of necrotic cells. X. Chen and colleague 50 designed a nanoplatform to co-deliver small-molecular drugs and siRNA for cancer therapy, by transplanting the distinct property of Zn-DPA covered by hyaluronic acid, which owned low toxicity and specific accumulation.

#### Co-encapsulation without drug interaction

The combination of delivering nucleic acid and small-molecular drugs always were regarded as a challenging barrier due to the defined differences of the two types of drugs, especially in their physicochemical properties, including the molecular weight, electrical properties, hydrophilicity or hydrophobicity, and metabolic stability, all of which greatly affect their biodistribution and pharmacokinetic properties 17, 51, 52. Most recent strategies were developed based on these differences and often the best choice was introducing a module for loading the chemotherapeutic agent into the existing carrier for nucleic acid drug. In addition, some vectors were specifically developed to meet the demand of both drugs and nucleic acids.

##### Regular distribution

**Figure 6:**
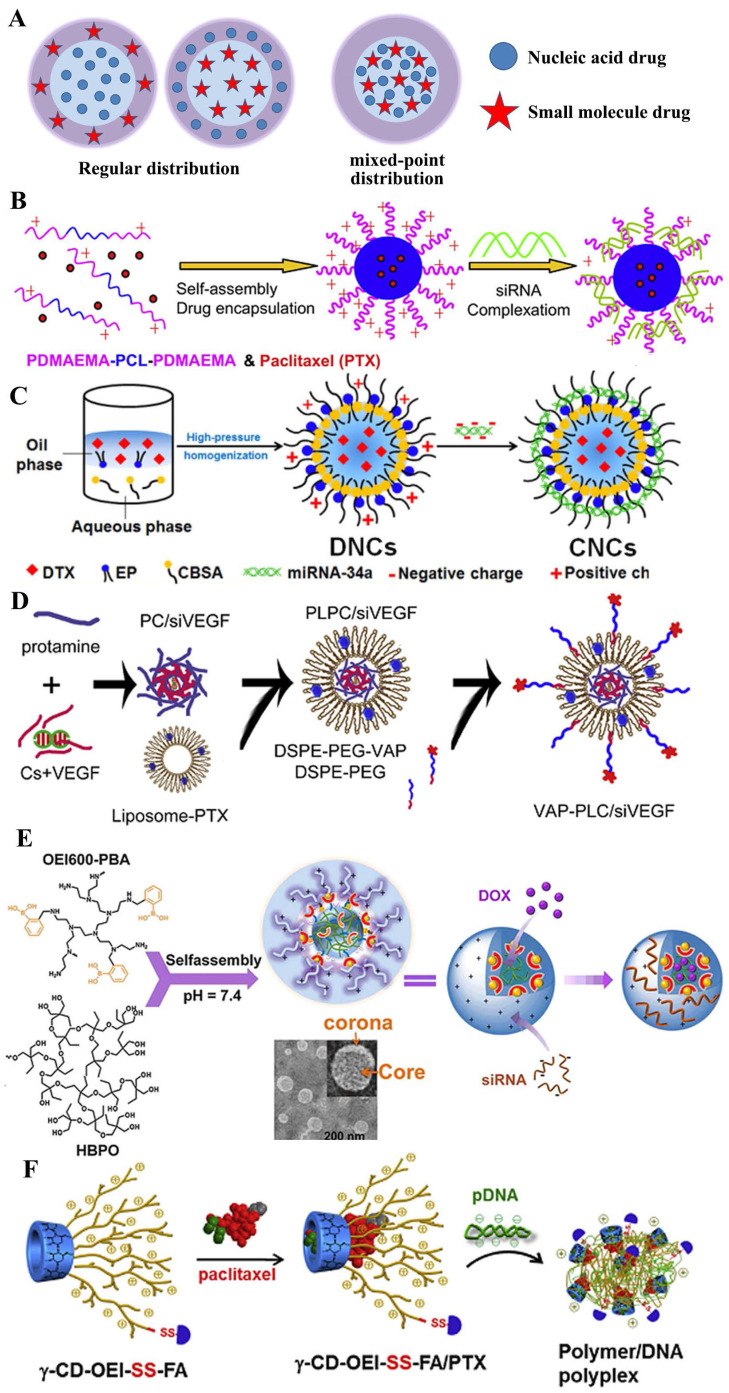
A) Structural illustration of co-encapsulation without drug interaction. Regular distribution: B) cationic PDMAEMA-PCL-PDMAEMA triblock copolymers to co-deliver paclitaxel and siRNA 53; C) core-shell nanocarriers to co-deliver DTX and miRNA-34a 54 and D) targeted core-shell type nanoparticles for the co-delivery of paclitaxel and siRNA 55. Mix-point distribution: E) nano-assembled HBPO(OEI600-PBA)_10_ for the co-delivery of DOX and siRNA 56; F) γ-cyclodextrin and γ-CD-OEI-SS-FA co-deliver paclitaxel and p53 gene for anticancer therapy 57.

In recent decades, gene therapy has been hailed as an important weapon for combating a variety of diseases, especially cancer. Thence, a variety of materials typified by cationic carriers have been developed to overcome physiological barriers, such as systemic stability, cellular targetability, immunogenicity and safety, for the delivery of genetic drugs, primarily including polyplexes and lipoplexes formed by cationic polymers or lipids with nucleic acids 51, 58, 59. Much of the research on the respective gene and drug systems provided positive direct principles for designing the co-delivery nanocarriers. Coincidentally, most hydrophobic drugs for cancer chemotherapy are easily loaded into the hydrophobic area of polyplexes or lipoplexes and the hydrophilic area carries the nucleic drugs through electrostatic interaction, which results in the layered or regular distribution in the entire system and the two drugs often appear to a serial release in the focus of infection (**Figure 6A**). The sequential release contributes to the collaboration of two drugs with different target factors, especially under the second and the fourth mechanism (as shown in **Figure 2 II and IV**).

In the earlier period, Tamara Minko et al. 60 developed a cationic liposome prepared from DOTAP to co-delivery DOX and two siRNAs targeted to MRP1 mRNA (pump resistance) and Bcl2 mRNA (non-pump resistance), respectively. In the simple co-delivery liposome, the siRNAs absorbed on the outer layer tended to release firstly to knocked the expression of MRP1 and Bcl2 that were related to muti-drug resistant, which paved for the later release of DOX and reduced its efflux. Thus, the system led to the efficient induction of apoptosis and killing of MDR lung cancer cells to a level that cannot be achieved by separate treatment with an anti-cancer drug or siRNA alone. As the manner shown in **Figure 6B**, based on PDMAEMA-PCL-PDMAEMA triblock copolymers, Zhong and his co-workers 53 constructed the biodegradable cationic micelles to co-deliver siVEGF and paclitaxel into PC-3 cancer cells, which revealed a further improved apoptosis efficiency. In addition to the organic vehicle based on other similar materials, like linear adamantane-terminated octadecane (C18-AD) and Tris(2-aminoethyl) amine-attached β-cyclodextrin-centered hyperbranched polyglycerol (CD-HPG-TAEA) 46, mPEG-PCL-graft-PDMAEMA 61, PLA-b-PDMAEMA 62 and PEI-β-cyclodextrin 63, various inorganic materials also were synthesized for co-delivering two drugs 64. By functionalizing the surface of mesoporous silica nanoparticles with a phosphonate group, it may be possible to electrostatically bind DOX to the porous interior, where the drug would be released through acidification of the media under both abiotic and biotic conditions. Hence, it was developed to co-deliver nucleic drugs (Pgp siRNA or p53 gene) and DOX with the modification of PEI 65, 66, PDMAEMA/PMPDSAH 67, respectively. Similarly, functionalized magnetic graphene nanoparticle (CMG) 68, QDs 69 and Se nanoparticles 70 platform was used for tumor treatment.

However, the cationic nanocarriers with poor security often remain in the in vitro evaluation stage and couldn't meet the needs of in vivo delivery. So as to improve the stability, the addition of PEG on the outer of nanocarriers became fashionable for system construction, such as mPEG_45_-b-PCL_80_-b-PPEEA_10_ for paclitaxel and siPlk1 71, PEG-PLL-PLLeu co-delivering docetaxel and Bcl-2 siRNA 72 or cSLN for co-delivering of paclitaxel and siMCL1 73 for better anti-cancer treatment. Cationic nanocarriers coated by anionic materials also could prolong the circulation time of co-delivery systems. Based on this, Zhang et al. 54 co-delivered miRNA-34a and docetaxel with core-shell nanoparticles for the metastatic breast cancer therapy (**Figure 6C**). Similarly, Nie et al. 74 designed an ε-polylysine cationic co-polymer to efficiently take up negatively charged si-HIF1a on the surface and encapsulate gemcitabine at the hydrophilic core, then further coating with PEGylated lipid bilayer to reverse the surface charge, which demonstrated excellent ability to inhibit tumor metastasis in orthotopic tumor models.

The problem always, the progress on the road. The PEG dilemma dragged the hind legs of the greatest effect of co-administration, and in order to circumvent these salient weak points, two main strategies were developed, one of which was to introduce the targeting ligands, such as the modified synthetic analog of Luteinizing Hormone-Releasing Hormone (LHRH) decapeptide 75, hyaluronic acid 76, TAT 77 and folic acid 78. Paula T. Hammond and co-workers 79 constructed a layer-by-layer nanoparticle (ie. CML/PLA/siRNA/PLA/HA) to co-deliver siRNA and chemotherapeutic drug to treat the triple negative breast cancer. In addition, the other strategy to overcome the PEG dilemma is the modification of a cleavable PEG. Li et al. 80 designed the nanoparticle to co-deliver PTX and siRNA, which consisted of the pH-responsive core, the cationic shell, and the matrix metalloproteinase (MMP)-cleavable PEG corona conjugated via a peptide linker. Once in tumor-microenvironment, the responsive shedding of PEG would contribute into the cellular uptake of two drugs. Similar systems also structured in other works, such as pH-trigger 66 and redox-sensitive 81 dissociation of protective outer layer. Chen et al. 82 developed a stepwise cleavable composite lipid nanocarrier (PTX/miR124-NP) composed of calcium phosphate to co-deliver PTX and miR124. It exhibited a superior ability to respond to the tumor microenvironment, in which the surface PEG layer was shed in the mildly acidic environment of the tumor tissues and the exposed oligomeric hyaluronic acid could facilitate the cellular uptake by targeting the CD44 receptor to the surface of the cancer cells. Once into cells, o-HA would detach from the core due to the reduction of disulfide bonds by glutathione and to inhibit tumor metastasis. PTX and miR124 were then released sequentially and exerted synergistic anti-tumor effects by reversing the process of the epithelial-mesenchymal transition in MDA-MB 231 cells.

Overall, small molecule drugs and nucleic acid drugs disperse in nanocarrier by a layered or regular manner, but nucleic acid drugs absorbed in the outer layer mostly, which is unfavorable for its protection in the process of circulation in vivo. A better method to avoid this shortcoming is encapsulate it into inner layer, such as the nanoparticles of multi-layers 83-85 or core-shell 86. As **Figure 6D** shown, Feng et al. 55 successfully constructed a core-shell nanoparticle to co-deliver PTX and siVEGF for the inhibition of breast cancer. In this study, siRNA was encapsulated in the negative-charged core to form the ternary complex composed of siRNA, chondroitin sulfate (CS) and protamine, which was coated by vapreotide-PEG modified cationic liposome. Meaningfully, the VAP-PCL/siVEGF obtained a stronger inhibition of tumor growth and better safety compared to the formulation with the single one drug.

##### Mixed-point distribution

Generally, during the construction of the vector, nucleic acid drugs and small molecule drugs are added sequentially and specifically loaded into the hydrophilic area and hydrophobic area or holes, respectively, so they appear to a layered or regular distribution in the co-nanocarriers. Sometimes, the boundary of the hydrophilic/hydrophobic or positive/negative area is not obvious and the areas tend to a state of staggered mixing distribution. Hence, the two drugs also co-loaded in the manner of “mixed-point distribution”, like the bright or dark stars dotted in the night sky. Compared to the system of layered or regular distribution, it often releases two drugs almost simultaneously and it is helpful to enhance the collaboration of two drugs with the same one target factor, especially under the first mechanism (as shown in** Figure 2I**).

Patil et al. 87 fabricated biotin-PEG-functionalized PLGA nanocarriers loaded with PTX and siRNA targeting multidrug resistance gene 1 (MDR1). The NPs were prepared by the dual emulsion method whereby the mixture of the PEI/siRNA complex and PTX was encapsulated by the PLGA and PLA layer. Additional results indicated that these dual-agent loaded nanocarriers were significantly more cytotoxic, suggesting that silencing MDR1 expression increased PTX accumulation in drug resistant JC cells and PTX/P-gp siRNA-loaded nanocarriers inhibited significant tumor growth than that loaded with PTX only. However, the double-emulsion solvent evaporation has failed in effectively removing the organic solvent, which limited its medical application. Furthermore, the supercritical CO_2_ technology has been employed for drug nanocarriers due to its mild operating conditions, lower solvent residue and lower cost 88. Chen et al. 89 fabricated the siRNA-PTX-CMPs by a supercritical process to co-load siP-gp and PTX for the treatment of drug-resistance Bcap-37 cells. The chitosan NPs containing siRNA prepared by ionic gelation and PTX were as core materials and were followed by encapsulation with poly (L-lactide)-poly (ethylene glycol)-poly(L-lactide) triblock copolymer (PLLA-PEG-PLLA) as a shell material. Furthermore, the results demonstrated that the siRNA-PTX-CMPs achieved a much better anti-tumor effect than the CMP encapsulated with PTX alone.

In the development of drugs, the complexity of preparation is often not favored, like the double-emulsion solvent evaporation or its improved proposals. People tend to develop a direct hybrid approach for the nanomedicine with two drugs. Deng et al. 90 constructed the nano-complexes by the one-step mixing method, which prepared using hyaluronic acid and chitosan that simultaneously encapsulate positive charged doxorubicin and negative charged miR-34a mimics, which not only resulted in an effective reduction of drug resistance and side effects of DOX, but also enhanced the therapeutic outcome of DOX, finally obtained the simultaneous inhibition of tumor growth and migration. Similarly, Jia et al. developed a hyperbranched-hyperbranched polymeric (HBPO(OEI600-PBA)_10_) nano-assembly with pH-dependent stability to co-deliver antitumor DOX and autophagy-inhibitory Beclin1 siRNA, which could silence the Becline1 expression and thereby suppress the DOX-induced cellular autophagy, leading to remarkably enhanced in vitro and in vivo antitumor efficacy 56, as shown in **Figure 6E**. In addition, various co-deliver system of mixed-point distribution were developed, such as Tf-modified co-encapsulated DOX- and pEGFP-loaded SLN for targeting lung cancer 91, the first use of nanoscale metal-organic frameworks (UiO-NMOFs) for the co-delivery of cisplatin and MDR gene-silencing siRNAs (Bcl-2, P-glycoprotein and survivin) to enhance therapeutic efficacy 92 and γ-cyclodextrin and multiple oligoethylenimine arms with folic acid (γ-CD-OEI-SS-FA) co-deliver paclitaxel PTX and p53 gene for potential cancer therapy 57** (Figure 6F)**.

#### Drug-carrier conjugates delivering drug (DCDD)

The functionalization of nanocarriers has been exploited in the last two decades. Actually, the synergic bioactivities of gene delivery systems have been increasingly highlighted by covalently inserting the small-molecular drugs into materials. By the stimuli-broken or non-broken junctions 96, 97, these modified materials could not only transport the nucleic acids into targeted sites but also sequentially produce the synergy effects, which reduced the leakage of drugs in the delivery process.

**Figure 7:**
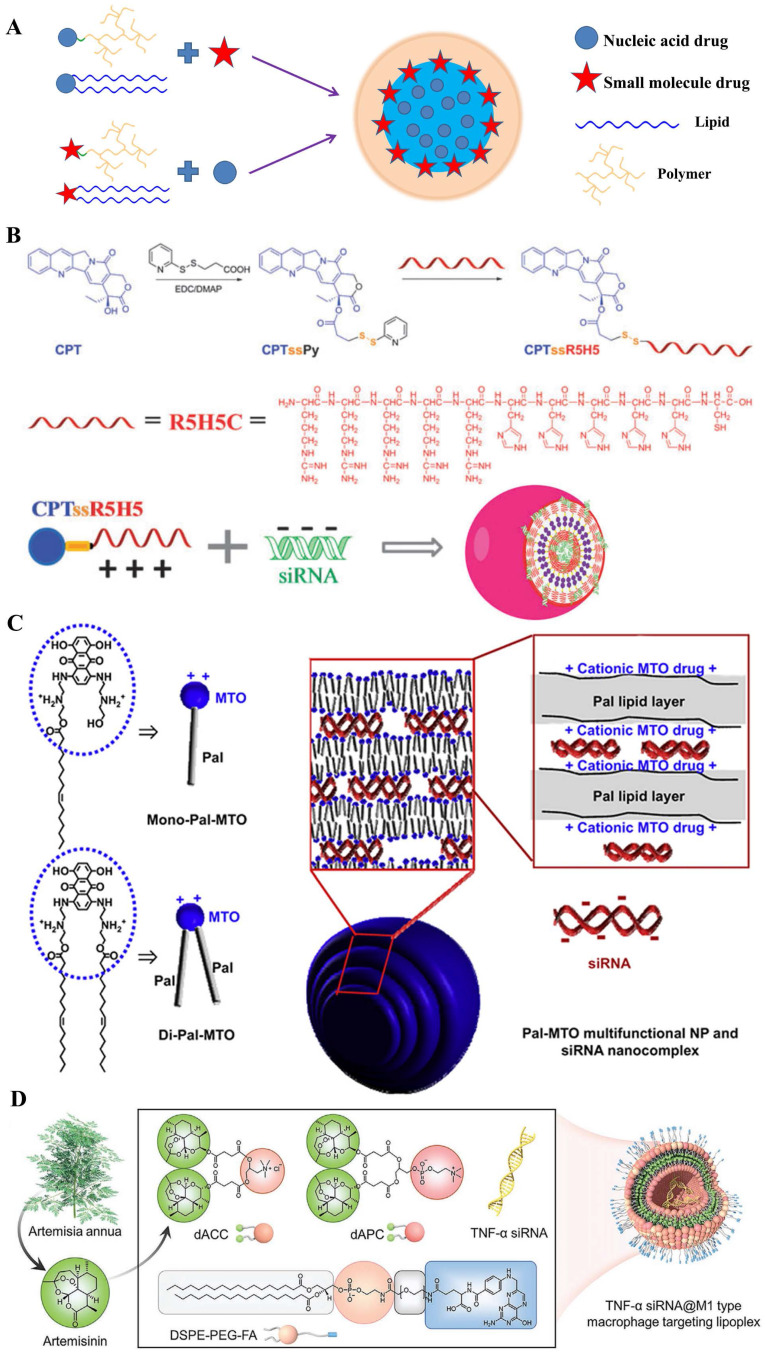
A) Structural illustration of co-encapsulation with prodrug-based carriers; B) Co-delivery system CPTssR5H5 to deliver CPT and MAP3K7-targeted siRNA simultaneously to MDR cancer cells for enhanced chemotherapy 93; C) Pal-MTO and siRNA nanocomplexes where cationic surface charges of MTO moieties of Pal-MTO could allow electrostatic interaction with siRNA. 94; D) Schematic illustration of amphipathic artesunate prodrug-hydrogel lipoplexes delivering TNF-α siRNA for rheumatoid arthritis therapy 95.

The important one is delivering genes by the materials comprised of small-molecular drugs. Nanoparticles for delivering gene drugs have been emerged over the last decades, where the auxiliary material was designed for biosafety and biocompatibility 98-100. Moreover, recent studies have covalently inserted several anticancer drugs into delivery materials 101-106, which was aimed to realize the bio-functionalization of carriers, thus, promoting the synergistic effect 107-112. In general, the hydrophobic anticancer small-molecular drugs (e.g. doxorubicin 113, 114, camptothecin 93, oleic acid 115 etc.) were grafted onto hydrophilic marcomolecules which could bind to gene. Three subdivisions were classified by the type of gene delivery systems, for instance, polysaccharide-modified macromolecules, cationic polymers, lipid-based nanoparticles and so on (**Figure 7A**). In addition, nucleic acids could take as a carrier for chemotherapy drugs delivery. According to the kinds of delivery materials and encapsulation methods, they were classified and detailed discussed respectively.

##### Polysaccharide-derived prodrug

Natural polysaccharides have been regarded as the ideal materials for construing drugs delivery systems 116. An unexpected result may occur that small-molecular drugs were jointed into their chemical skeletons. H.L. Jiang and colleague designed a hierarchical targeted delivery system loading siRNA and lonidamine (LND) drug sequentially to tumor cells and mitochondria 117. As known, LND could inhibit hexokinase and directly impact mitochondrial adenine nucleotide translocase to trigger the opening of the mitochondrial permeability transition pore and then induce tumor cell apoptosis 118. In this nanostructures, two parts were defined as TCPL and PPF. The former was obtained by linking LND and TPP (mitochondria targeting ligand) to the chitosan-g-PEI polymer with Schiff base bonds; and the latter is the PEG-b-poly (acrylic acid) copolymer comprised of folic acid. Through an easy mixture preparation, the hybrid nanoparticles could enhance the tumor cell uptake and promote the co-drugs release. These results showed that treated with the nanomedicine, more cytochrome C (up to two times) were released from mitochondria in Hela cell cytosols; moreover, the quantity of both activated caspase 9 and caspase 3 was sharply increased in HeLa cells; furthermore, a significant decrease (down to ~20%) of Bcl-2 mRNA and Bcl-2 protein were also observed by the assays of qPCR and western blotting. All these results demonstrated that the co-delivery of effective hierarchical system could synergistically activate mitochondria-mediated apoptosis. Similarly, T. Chen and colleague embedded the adriamycin (ADM) into the dextran-graft-PEI (DEX-g-PEI) copolymer, which delivered the plasmid for osteosarcoma disease 113. This research presented that the DEX-ADM-PEI nanoparticles could efficiently deliver both pEGFP-N1 and ADM to osteosarcoma cells with low cytotoxicity. In addition, J. Li doxorubicin with amantadine-modification was through the “host-guest” hydrophobic force to be linked to the polyethylenimine-co-cyclodextrins (PEI-co-CD) polymers 119, where plasmid DNA was encapsulated to form the hybrid co-delivery nanoparticles (denoted as SNP). These results displayed that a remarkable decrease of cytotoxicity was seen in murine melanoma B16F10 cells; moreover, the intracellular release from nano-system dissociation and siRNA transfection were simultaneously observed; furthermore, the formation inhibited the tumor growth in vivo.

##### Cationic polymers-derived prodrug

Prodrug-like gene carriers could be obtained by combination of hydrophobic drugs with cationic hydrophilic polymers 120, 121. In the research of G. Cheng and his colleagues 93, anticancer drug camptothecin (CPT) was conjugated to a hydrophilic cationic peptide (R5H5) using disulfide bond as a linker; then the synthesized material of CPTssR5H5 was developed in siRNA delivery for MDR cancer therapy **(Figure 7B)**. These co-delivery nanopolymers displayed a suitable parameter (size ~60 nm and zeta ~6 mV); moreover, CPTssR5H5 could carry MAP3K7 siRNA into MDA-MB-231 cells. Furthermore, the result of RT-PCR assay showed an important decrease (~30%) of MAP3K7 mRNA expression in MDA-MB-231 cells at siRNA concentrations of 100 nM. Compared with cationic peptide, another classic cationic hydrophilic polymer (PEI) was also polymerized by an AT1 receptor blocker drug named eprosartan (ES) for DNA delivery. Currently, many reports verified that AT1 receptor blockers (ARBs) exert beneficial effects on tumor progression, vascularization, and metastasis 122. J.P. Zhou and colleagues 123 designed a reticular eprosartan-g-PEI (ESP) copolymer mediated targeted ES drug and p53 gene co-delivery system. The ESP loading pDNA was a spheroidal particle shared a 150 nm in size and ~16 mV in zeta potential; in PANC-1 cells, the data of MTT assay showed almost non-cytotoxicity (Over 90% cell viability) at the concentration of ESP used dose. Subsequent assays implied that the nanomedicine could significantly inhibit the expression of Ang II-induced angiogenesis related gene VEGF, which the inhibition rate achieved 83%; moreover, anticancer efficacy in vivo displayed an important tumor growth inhibition efficacy and the final tumor volume was below 500 mm^3^, far less than that in other groups. All these results demonstrated it an effective co-delivery strategy for cancer therapy. Similarly, L. Huang and his colleague 124 polymerized the pharmacophores of Metformin into PEI polymers. On account of the anticancer efficacy of Metformin in many cancers, a co-delivery system named LPH-PolyMet was developed to human non-small-cell lung cancer (NSCLC) treatment. Several results displayed the LPH-PolyMet could high efficiently silence the expression of VEGF mRNA (down to 5%) in NSCLC tumor cell H460, and then induced apoptosis in about 40% of cells, which implied that PolyMet play dual roles as both a gene carrier and an antitumor drug to achieve combinational therapeutic efficacies against cancer.

##### Lipid-derived prodrug

There was a major of studies involved in amphiphilic where the drug was modified by hydrophilic or hydrophobic groups 125, 126, thus, several drug-inserted lipids could be reasonably applied in **DCDD**. Mitoxantrone (MTO), as an anticancer chemical drug with cationic property, was conjugated to hydrophobic palmitoleic acid to form the drug-modified lipid for delivering siRNA 94 (**Figure 7C**). These hybrid nanoparticles were ~220 nm in size and had a narrow PDI index. These results presented that it significantly down-regulated the expression of red fluorescence protein in B16F10-RFP cells (down to ~40%); moreover, antitumor assay in vivo displayed that these nanoparticles loading with Mcl-1-specific siRNA (siMcl-1) could be internalized into human KB tumor cells; and demonstrated a cooperative effect which reduced tumor size by 83.4%. In general, the only cationic drug-modified lipid was not satisfied for development of nucleic acids in clinic. Therefore, some prodrug-hybrid nanoparticles were designed for co-delivery with gene drugs. X. Zhang et al. 127 designed a dual-responsible camptothecin-poly(carboxybetaine) conjugate (PCB-CPT), which could effectively release the CPT based pH and esterase-sensitive cleavage. With the cationic lipid named DDAB, the nano-complexes loaded with siRNA (siPlk1) were prepared to targeting the polo-like kinase 1 in HeLa cells. Results showed that no evident initial burst release was observed in pH 7.4 medium; moreover, the release rate of CPT drugs was accelerated to ~57% with 120 h in a sustained manner at the acidic conditions. Additionally, the amphipathic cisplatin prodrug has also been integrated into the nanoparticles with siRNA co-delivery. Chosen the PLGA-PEG and the cationic lipid named as G0-C14, O.C. Farokhzada and colleague 128 constructed the co-delivery system which simultaneously encapsulated cisplatin prodrug and siRNAs against REV1/REV3L to promote chemo-sensitivity. The hybrid nanoparticles achieved greater than 95% luciferase silence in luciferase-expressing HeLa-derived cells (Dual-Luc HeLa), while no cytotoxicity was evidently observed. Similarly, Li et al 95 synthetized two novel artemisinin derivatives (dAPC and dACC) which possessed mimic phospholipids and cationic lipids, respectively (**Figure 7D**). Then, they prepared a cationic multicomponent drug-embedded liposome and the TNF-α siRNA/artemisinin co-delivery nano-microplex (MTAsi@MG) by immobilization of TNF-α siRNA/lipoplex on porous microfluidic HA microspheres. The results indicated that it might be one potential gene/drug co-delivery systems for rheumatoid arthritis therapy.

**Figure 8:**
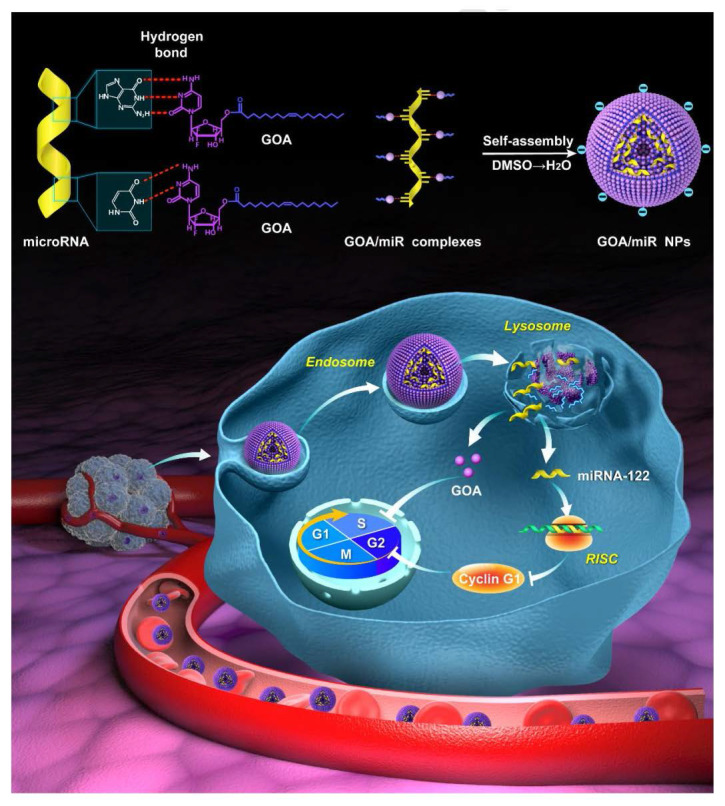
Structural illustration of co-deliver microRNA with gemcitabine-oleic acid conjugate (GOA) based carriers to inhibit the growth of tumor 129.

Gemcitabine, a widely used chemotherapeutic drug, also be designed as a prodrug carrier because the cytosine of gemcitabine could bind to miRNA by hydrogen bond interaction of nucleobases. As shown in **Figure 8**, Zhang et al. 129 synthesized an amphiphilic gemcitabine prodrug (gemcitabine-oleic acid conjugate, GOA) and it was used to bind miRNAs with hydrogen bond forces between nucleobases in dimethyl sulfoxide via a denaturation-annealing procedure. The results indicated that the GOA/miR nanoparticles would be efficiently internalized into cancer cells after intravenous administration and possessed significant tumor growth inhibition effects.

##### Nucleic acids-derived prodrug

**Figure 9:**
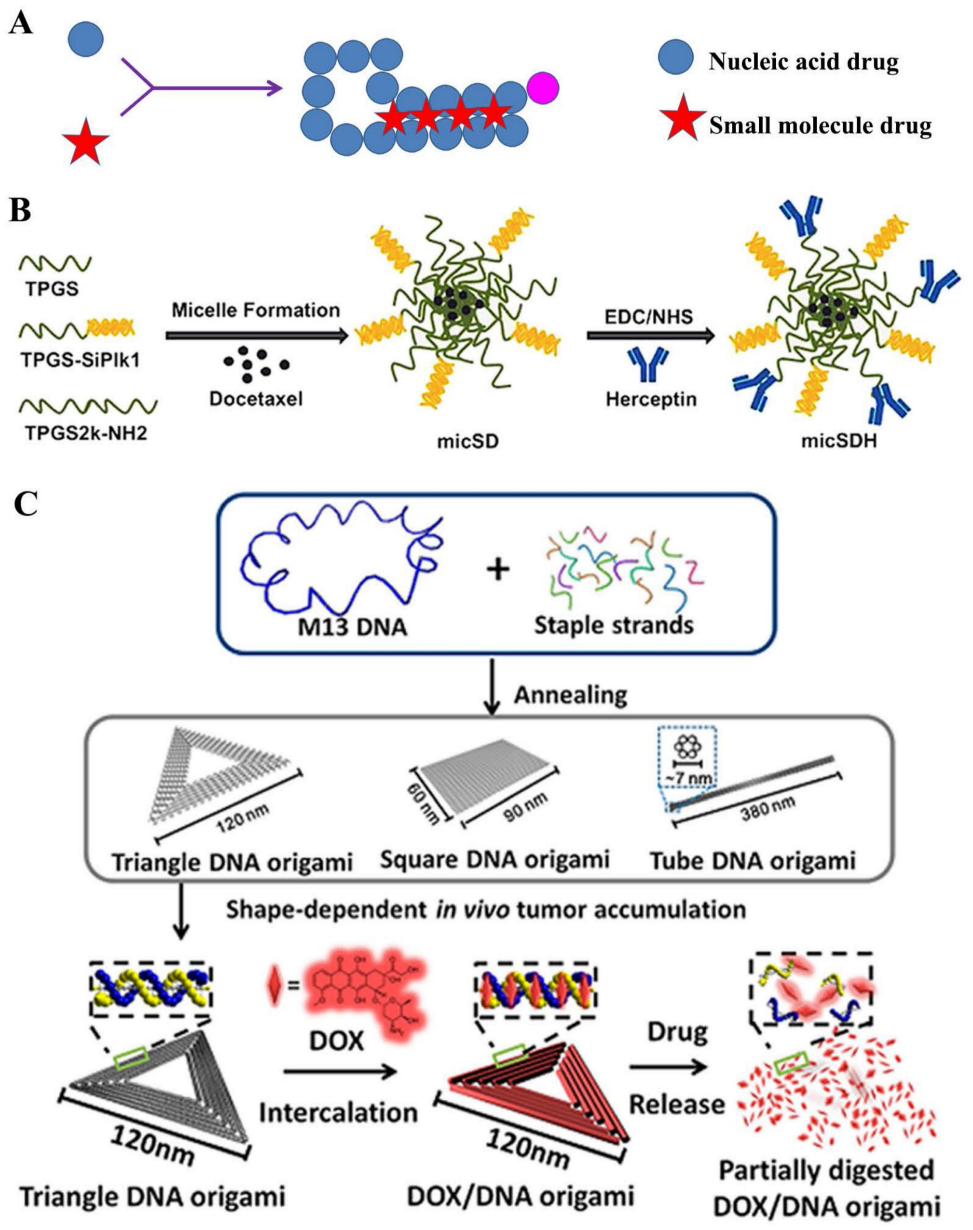
A) Structural illustration of nucleic acids-derived prodrug; B) Formulation of the docetaxel loaded TPGSesiPlk1/TPGS micelles (micSD) 130; C) Schematic design of the triangle-shaped DNA origami for doxorubicin intercalation 131.

Theoretically, siRNA was apt to be degraded by a series of ribozymes, leading to the instability in the transport process 132. Thus, the siRNA conjugates could ameliorate this dilemma 133-135; therein, amphiphilic siRNA-lipids could as a carrier or auxiliary for co-delivering small-molecular drugs 136 (**Figure 9A**). S.S. Feng and his colleague 130 constructed a co-delivery nanomedicine of docetaxel which was comprised of TPGS-siRNA conjugates and herceptin-conjugated vitamin E TPGS for tumor treatment (**Figure 9B**). In this system, siRNA conjugate, which was linked by the redox-responsive disulfide, was targeted for the polo-like kinase 1 (Plk1) protein. Results displayed that the ~190 nm micelles could release ~70% of docetaxel drugs in HeLa cells after 120 h; moreover, at the concentration of 125 nM, an important knockdown (88.8%) of Plk1 mRNA expression could be clearly observed. Interestingly, bis-siRNA-phospholipid conjugate played an auxiliary role in co-delivery of DOX drugs. In this study 137, the nanoparticles (siRNA-PCNPs) loading with DOX was composed of siRNA-phospholipids, cationic lipids DDAB and DSPE-PEG_2000_ fuse around PLGA for targeting Polo-like kinase 1 protein in HeLa cells. Dual functions of siRNA conjugates were designed that gene silence and as a component of nanoparticles to embed hydrophobic DOX drugs. Subsequent results showed that the nanoparticles importantly down-regulated Plk1 mRNA to ~30% in HeLa cells and induced 76.4% apoptosis cells, which effectively inhibited the tumor growth. Besides, siPlk1-phospholipids sharply enhanced the encapsulation of siPlk1 (three times), which was auxiliary for loading drugs.

Several double-stranded DNA or aptamer, among the nucleic acids, presented a relatively high stability to meet the in vivo drug delivery 45, 138-140. Therein, the small-molecular drug, such as DOX, intercalate into DNA base pairs, with a preference for GC/CG regions 141. H. Mok and colleague 142 synthesized multivalent aptamer-siRNA conjugates as material for co-delivering doxorubicin. This conjugate contained multiple mucin-1 aptamers and Bcl2-specific siRNA, which were attempted to transfect into mucin-1 overexpressing MCF-7 breast cancer cells for combination cancer therapy. Assay data displayed that the DOX-Apt-siRNA gradually released DOX≈80% during one day incubation; moreover, high cellular uptake was observed in both wild-type and MDR MCF-7 cells; furthermore, the conjugates loading with DOX presented two-fold higher of caspase-3/7 activity than that in free DOX group, which may exert important cytotoxic effects for MDR cancer therapy. Recently, DNA origami techniques provided a potential platform for novel drug delivery 143. Y. Du and his colleague 131 designed the triangular-shaped DNA origami vehicles to load DOX through intercalation. Therein, DNA origami carriers were obtained through the self-assembly between M13mp18 phage DNA the complementary DNA helper strands, where the doxorubicin was subsequently non-covalently intercalated into (**Figure 9C**). A series of assays showed that triangle DNA origami was ~120 nm, and loaded ~50% DOX; moreover, the release slowly progressed, which reached to ~20% under physiological conditions at 48 h. Furthermore, in cytotoxicity assay, these nanomedicines displayed an important antitumor efficacy in the MDA-MB-231-GFP cells. All these results implied that DNA origami had great potential in clinic for safely and efficiently delivering antitumor drugs. Similar strategy was also used for DOX co-delivery by microRNA 140.

## Conclusion and Future Direction

Recent developments in the nanotechnology field have provided avenues for the delightful nanomedicine-based combination anticancer therapy between nucleic acids and small-molecular drugs. This review covers new trends in combination therapy and discusses three key issues in detail: 1) different types of collaboration mechanisms; 2) categorized preparation methods; and 3) relatively little reported multiple delivery systems based on the combination of chemotherapeutic and gene agents. So as to achieve the satisfied therapeutic effects of anticancer therapy, three approaches to combination therapy were concisely concluded as follows: 1) different drugs in one nano-system (all-in-one) could synchronously escort the drugs into the same targeted sites; 2) the multiple stimuli-responsive nanocarriers of co-delivery with chemotherapy and gene drugs could be constructed for promoting their effective uptake and release in tumor cells; 3) co-administration of different drugs in separate nanocarriers could verify the kinetics of their pharmacologic activity, respectively (**Table 1**).

As the aforementioned descriptions, it was observed that the strategy of “co-assembling drugs” in independent zones was mainly reported in the field of the nanomedicine-based combining therapy, which generally presented an amphiphilic nano-system where anticancer drug was encapsulated in hydrophobic sections; while the design of drug-carrier conjugates delivering drug (DCDD) would be increasingly potential in synergistic treatment of cancers. By the stimuli-broken bond as linkers, these prodrug-modified carriers could not only effectively deliver genes into targeted cells but also sequentially produce the synergy effects of tumor treatment. Besides, the “corporate drug pairs (CDP)” suggested both gene and anticancer drugs were encapsulated in the core area of the nanocarriers, which should be considered as a promising approach where all the drugs had the capability of resistance against degradation and synchronous release.

The combined delivery strategy could importantly enhance anticancer effects by several aspects, for instance, accumulating drugs to tumor sites in vivo, surmounting the multiple drug resistance (MDR) or inducing different apoptosis pathways etc. However, there were still numerous technical barriers that hampered the development of the combination approach in the clinic. Therein, a deeper understanding of the nature of tumor heterogeneity should be the prerequisite; besides, the clear relationship was also needed between the pharmacodynamic response and associated toxicity/immune response in the simultaneous administration of gene and chemo drugs. In addition, the high developmental costs of designing the combined-complex systems were still an extreme challenge in the clinical development. Therefore, as the potential strategy, the combined nanomedicines need to overcome a variety of efforts to further clinical application between small-molecular drugs and nucleic acids drugs.

## Figures and Tables

**Table 1:** Summary of representative small molecule drug-nucleic acid drug combinations.

Drug encapsulation	Delivery method		Synergistic mechanism	Small molecule drug	Nucleic acid drug	Cell line	Overcome problems	Ref
**“1+1”** **type**	**Separate systems**	**Different carriers**	IV	Doxorubicin	TNFα pDNA	Neuro2A	Proliferation	21
			IV	Paclitaxel	Cyclooxygenase-2 siRNA	HepG2 U87MG	Hypoxia-Induced MDR	19
			IV	Doxorubicin	Bcl-2 siRNA	HeLa	MDR	23
		**Same carrier**	IV	Doxorubicin	P-gp siRNA	MCF-7/A	MDR	24
			IV	Doxorubicin	Bcl-2; MDR1 siRNA	HeLa; MCF-7	MDR	26
**“2 in 1” type**	**“Corporate drug pairs (CDP)”** **encapsulated** **in core**	**Electrostatic interaction**	III	Benzethonium	Bcl-2 siRNA	MDA-MB231 MRC-5 NIH3T3	Aggressive MDR	37
			II	Gemcitabine	antisense- miRNA-21	Hep3B; HepG2	ProliferationTotoxicity	39
			II	Doxorubicin	IL17RB siRNA	MDA-MB361	Apoptosis; migration	41
		**Chemical coupling**	IV	Doxorubicin	DNA aptamer sgc8	CEM Ramos	Resistant to nuclease degradation	38
			IV	5-FU	MT-II and LIB aptamer	HCT116	Photo- controllable release	45
		**Coordination bond**	III	Gemcitabine	VEGF siRNA	H460	Anti-growth	39
			III	Zn^2+^-DPA	Luciferase siRNA	4T1	Targeting	50
	**Co-encapsulation without drug interaction**	**Regular distribution**	I	Paclitaxel	VEGF siRNA	PC-3	Apoptosis and anti-growth	53
			IV	Docetaxel	miRNA-34a	4T1	Apoptosis	54
			II	Gemcitabine	HIF1α siRNA	Panc-1	Proliferation; MDR	74
			II	Paclitaxel	MCL1 siRNA	MDA-MB231	Proliferation; MDR	73
			III	Camptothecin	Raf-1 siRNA	C6 rat glioma	Proliferation and apoptosis	93
		**Mix-point distribution**	II	Doxorubicin	Beclin1 siRNA	HeLa	Apoptosis	56
			I	Paclitaxel	P53 pDNA	A549	Apoptosis	57
			IV	Cisplatin	P-gp siRNA	SKOV-3	MDR	92
	**Drug-carrier conjugates delivering drug (DCDD)**	**Small** **molecule drug-based carrier**	II	Mitoxantron	MCL1 siRNA	B16-F10	Apoptosis	94
			I	Captopril	GFP, VEGF siRNA	MDA-MB435	Anti-angiogenesis	105
			II	Porphyrin, Docetaxel	MMP-9 shRNA	HNE-1	PDT; apoptosis	120
			I	Eprosartan	P53 pDNA	PANC-1	Anti-angiogenesis	123
			III	Metformin	VEGF siRNA	H460 1205Lu	Apoptosis; proliferation	124
			IV	Camptothecin	PLK1 siRNA	HeLa	MDR	127
			IV	Cisplatin	REV1, REV3L siRNA	MDA-MB231	MDR	128
			II	Pheophorbide A	PD-L1 siRNA	B16-F10	Diagnosis and treatment	144
		**Nucleic acid drug-based carrier**	II	Doxorubicin	pORF-hTRAIL pDNA	Bel-7402	Apoptosis; proliferation	114
			II	Doxorubicin	PLK1 siRNA	HeLa SK-BR-3	Apoptosis; proliferation	130, 137
			III	Doxorubicin	PLK1 siRNA miRNA-21	HeLa	Apoptosis and anti-growth	140
